# Rethinking the Fertility Transition in Rural Aragón (Spain) Using Height Data

**DOI:** 10.3390/ijerph18168338

**Published:** 2021-08-06

**Authors:** Francisco J. Marco-Gracia, Margarita López-Antón

**Affiliations:** 1 Department of Applied Economics and Economic History, Universidad de Zaragoza and Instituto Agroalimentario de Aragón, IA2 (UNIZAR-CITA), 50005 Zaragoza, Spain; 2 Business Department, Faculty of Business and Economics, Universitat Autònoma de Barcelona (UAB), 08193 Bellaterra, Spain; margarita.lopez@uab.cat

**Keywords:** fertility transition, biological well-being, health, height, intergenerational

## Abstract

Based on an analysis of the life trajectories of 2510 conscripts and their families from a Spanish rural area in the period 1835–1977, this paper studies the development of the fertility transition in relation to height using bivariate analyses. The use of heights is an innovative perspective of delving into the fertility transition and social transformation entailed. The results confirm that the men with a low level of biological well-being (related to low socio-economic groups) were those who started to control their fertility, perhaps due to the effect that increased average family size had on their budget. The children of individuals who controlled their fertility were taller than the children of other families. Therefore, the children of parents who controlled their fertility experienced the largest intergenerational increase in height (approximately 50% higher). This increase could be due to the consequence of a greater investment in children (Becker’s hypothesis) or a greater availability of resources for the whole family (resource dilution hypothesis).

## 1. Introduction

The demographic transition refers to the process where the population of the majority of countries has shifted from a context of high fertility and mortality to a new state of low fertility and mortality [1]. The fertility transition, meanwhile, is the process of fertility decline, which is part of the demographic transition. The first proposals to theorise the demographic transition were made in the first half of the twentieth century [2,3], and since then, thousands of studies have been conducted on this phenomenon (for an overview of the process: [4,5]). The demographic transition is one of the most important events to have happened to humankind, along with the Neolithic revolution and the industrial revolution, since it represents a change from the general behaviour of previous millennia. With the transition, societies also changed their mentality regarding sexuality, life and death [6]. The consequences have been felt in economic structures and populations. The fertility transition was the cause of the ageing of populations in several countries and has enabled married women nearly continuous access to the labour market, as their work is not interrupted by continuous childbirth and breastfeeding periods [7]. Moreover, the fertility transition has been one of the main contributors to the increase in GDP per capita since the end of the nineteenth century, particularly in its early stages [8,9,10,11,12]. We cannot understand the evolution of western society in recent centuries without taking into account the process of demographic transition.

This article focuses on the fertility transition in rural Spain, analysing how the reduction of marital fertility developed. Contraception was legally forbidden in Spain for almost the entire period of analysis and was finally decriminalised in 1978. As a result, Spanish women, especially rural women, found it difficult to access any type of synthetic contraceptive [13,14]. Therefore, fertility control was based on freely available natural methods such as *coitus interruptus,* abstinence and, to a lesser extent, vaginal douching, pessaries and sponges [14,15,16]. With respect to controlling fertility, two main strategies were applied: 1. Stopping; detaining marital fertility once the number of children that families considered sufficient had been reached; 2. Spacing; extending birth intervals to reduce fertility [17]. There is consensus that stopping is the fertility control strategy associated with the fertility transition and the most commonly used in the period in western countries [1,7,18,19,20,21,22,23,24]. Therefore, based on the methodology developed by Alberto Sanz-Gimeno and Fernando González-Quiñones [23], which is explained below, this study identifies the families who controlled their marital fertility using stopping as representatives of the individuals who controlled their fecundity during this period. Families that used stopping are easily identifiable and clearly associated with fertility control.

For this article, we understand biological well-being at the individual level as one of the dimensions of well-being linked to nutrition and health. Thus, most anthropometric, such as height or body mass index, and demographic, such as mortality, indicators are linked to biological well-being. When approaching biological well-being in this study from height, we are taking into account the net nutritional status resulting from food consumption, but also the wear and tear caused by the diseases experienced, the workload and the basal metabolism during the years of growth. In other words, it would be the stock of health during the development/growth period. In the study area, historically, height was largely affected by environmental and nutritional factors in the uterus and during childhood.

Several studies suggest that fertility control was started by groups with a higher socio-economic status [25,26]. This article analyses the relationship between biological well-being and the fertility transition in an agrarian area of Spain. We ask, was there a link between a higher level of biological well-being and the pioneering of the fertility transition in this area? The study also seeks to determine the consequences that the fertility control exercised by families had on the welfare of their children; was there a greater investment in their biological welfare when fertility was controlled? Finally, it seeks to analyse how socio-economic status and family size were linked to this process from the point of view of heights. Therefore, through the analysis of families that controlled their fertility, we aim to explore the relationship between family behavioural factors and improvements in biological well-being during the fertility transition.

We use height as a proxy for biological well-being. Numerous studies have shown that height is a good indicator of nutrition and epidemiological context during childhood and adolescence [27,28,29,30,31,32]. In recent decades, a large body of anthropometric research has demonstrated a close relationship between height and inequality in biological well-being [33,34,35,36,37,38,39,40,41]. In any case, we should not forget that height is mainly conditioned by genetics. Only a small fraction of growth is conditioned by other various factors such as nutrition, epidemiology or the environment [33,40,42,43,44,45,46].

This article uses a sample of 2510 conscripts born in 14 rural Spanish villages during the fertility transition and the previous decades. In the area of study, the fertility transition began at the turn of the twentieth century. There was a continuous fall in fertility levels, with the decline in the number of births during the 1930s being particularly important. Fertility continued to fall to below replacement levels in the last third of the twentieth century [17]. Families in the study area began to feel the drop in mortality from the 1860s onwards, although there was no sharp drop until the last decade of the nineteenth century [47].

The analysis uses height data corresponding to military conscripts at approximately 21 years of age and for whom we also have information about family trajectory. Unlike selection biases found in other countries, Spanish military recruitment records include all individuals of that generation. The existence of a universal recruitment system from the 1830s ensured that most recruits were measured, except for fugitives, some migrants and those who had died. Although several legal mechanisms existed between 1837 and 1936 to avoid compulsory military service, it is important to note that all of them were implemented after measurement [48,49,50,51]. All of the individuals in this study were required to undergo the first medical examination, from which almost all of the height records (92%) were extracted. The remaining 8% were obtained from the Historical Military Archive of Guadalajara, where copies of the files were deposited. Individuals rejected for military service because of their short height, or health problems were registered with the rest of the conscripts.

The family reconstitution method [52] was used based on the parish archives of baptisms, marriages and deaths for the 14 villages of the study. This source of complementary information has enabled us to confirm that all men who reached adult age were called up to conscription, and we have found no biases for any socio-economic groups. In any case, studies on stature may carry biases inherent in the military selection process itself that we cannot control for in the sample [53,54,55].

There is extensive knowledge about the evolution of height over time as an indicator of biological well-being. However, we know little about the relationship between this variable and the processes of demographic modernisation that have conditioned the development of living standards and inequality (and in some countries, still condition them as they are immersed in the process of demographic transition). The populations concerned undergo a process of social and economic change at both national and household levels. These processes can provide useful clues and ideas for identifying patterns in developing country populations and for designing appropriate public health and child nutrition policies. The study takes a long-term perspective of the rural context, allowing us to examine various in-depth stages of generational cohort analysis and the intergenerational transmission of well-being, taking into account the family socio-economic status. Hatton and Martin [56], in their pioneering paper on the relationship between fertility transition and height in Britain (1886–1938), based on the Boyd Orr survey, determined that height was strongly influenced by per capita income and family size. In fact, they concluded that the effect of the falling family size alone accounted for an increase of 1.6 centimetres so that 60% of the increase in heights was due (directly or indirectly) to the effects of falling family size. Ultimately, they found that fertility decline led to the rapid improvement in the health of children in the first half of the twentieth century. Previously, based on regional aggregate data for French conscripts between 1840 and 1911, Weir [57] determined that approximately 75% of the increases in height are associated with a reduction in marital fertility.

This article is innovative because, for the first time, it connects the process of fertility transition in rural Spain, through an analysis of fertility control strategies, with the study of the biological well-being of parents and children as measured by stature using longitudinal demographic data. This perspective allows us to rethink the transformation of society during the twentieth century and to understand the importance of fertility control for families with low standards of living. The results obtained help us to understand the importance of the fertility transition process in improving the living standards of the poorest groups, shedding new light on why the fertility transition process was a key pillar of per capita economic growth in the twentieth century. This study is ground-breaking in that it allows us to understand the importance of the fertility transition at the individual and family levels.

## 2. Area, Data and Methods

### 2.1. Area

**Figure 1:**
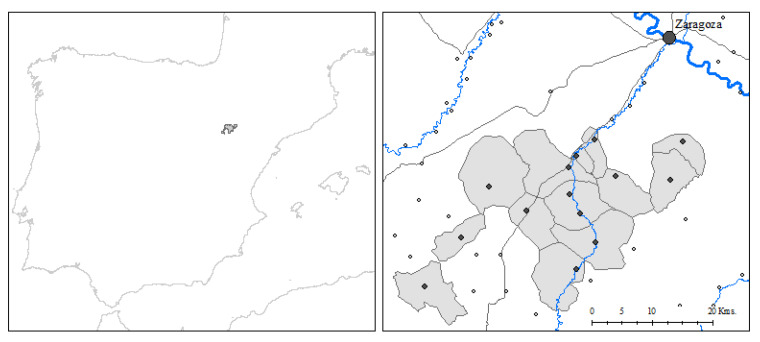
Area of study: Middle Huerva (Aragón, Spain) Note: Dark dots refer to the localities studied (except Zaragoza, the provincial capital) and the corresponding shaded areas to their municipal boundaries. As well as rivers (in blue) and main roads (grey), the map also depicts neighbouring villages (white dots).

The area of study is composed of 14 rural villages and is located in the region of Aragon, in the northeast of the Iberian Peninsula (see Figure 1). The villages of the study area are: Alfamén, Aylés, Botorrita, Codos, Cosuenda, Jaulín, Longares, Mezalocha, Mozota, Muel, Torrecilla de Valmadrid, Tosos, Valmadrid and Villanueva de Huerva. The area had a population of approximately 8000 inhabitants in 1860, 8200 in 1900, 10,700 in 1940 and 5600 in 1980 (see Table 1 for the distribution of the population by village). The border of the area is 19 km away from Zaragoza, the regional capital. This area is made up of a combination of plains and foothills (the initial mountains of the Iberian System) around the Huerva River.

The population mostly lived in nuclear households and was essentially devoted to agriculture (cereals and vineyards) and sheep grazing. Until the mid-twentieth century, 80 per cent of the male working population was engaged in the agricultural sector [17], where most of the population enjoyed living standards close to subsistence levels. Of the individuals engaged in agriculture, approximately 35–40 per cent were owners (generally smallholders), while the remaining 40–45 per cent were semi-landless and landless. Native shepherds made up approximately 7–8 per cent of the individuals, decreasing with the clearing of new land from the late nineteenth century onwards. Small artisans (blacksmiths, carpenters, etc.) were approximately seven per cent. The remaining 5–6 per cent was upper-class individuals (doctors, veterinarians, teachers, etc.) and other categories (such as bricklayers or military). Throughout the twentieth century, with economic modernisation, the arrival of tractors and other agricultural machinery, and the imposition of agrarian capitalism, the number of day-labourers needed in the agrarian tasks in the study area was reduced, so some of the landless and semi-landless had to migrate to the cities or become low-skilled workers in factories and other businesses such as construction.

All the agriculture in the area was in non-irrigated areas except for the land near the river Huerva, where fruit and vegetables could be cultivated. In 1947, ‘Las Torcas’ Reservoir was built in the municipality of Tosos and, from the 1970s onwards, irrigation infrastructures began to be developed, allowing agricultural production to be increased [58,59].

The average fertility was relatively stable at around 6–7 children per family; this lasted up to 1900 and declined rapidly thereafter, following the fertility transition. The study area was a high mortality region (only a little over half of the children survived to their fifth birthday). Mortality rates began to decline in the second half of the nineteenth century due to increasing living standards. Anthropometric evidence indicates that standards of living were low–the average male height was around 160 centimetres in mid-nineteenth-century, well below that of their European counterparts or their fellow Spaniards in other regions [60,61,62].

Aragon (the region) underwent a process of economic modernisation from the second half of the nineteenth century, which continued for most of the twentieth century despite economic and social shocks [63,64]. This economic modernisation was particularly prominent in the regional capital [65]. The privileged location of the Ebro Valley, close to the highly industrialised regions of Catalonia, the Basque Country and Valencia, and the proximity to the Spanish capital, Madrid, favoured the economic development of the valley. In 1857, Aragon was not an important industrial area in comparison to other Spanish regions, ranking ninth out of fourteen in terms of industrialisation [66,67]. The first stages of economic modernisation coincided with the first wave of globalisation and, in this region, this was linked to the development of the sugar industry [64,68,69]. The Spanish Civil War constituted a strong negative shock to Aragon’s economic modernisation, from which it did not recover until the 1960s [64]. Most of the industries were located in the Ebro Valley, relatively close to the area of study, which may have favoured rural-urban migration [70]. When comparing Aragon with the rest of the Spanish regions, on the basis of other indicators such as per capita income, productivity, labour productivity, welfare and inequality, Aragon had a slightly better situation than the Spanish average [71,72,73]. The rural areas of the Ebro Valley specialised in the agricultural products for the Spanish domestic market, such as cereals, sugar beet and sheep meat.

### 2.2. Data

The following three types of data have been used: 1. height data drawn from military conscription records; 2. individual demographic data drawn from parish registers (up to 1950), surveys (from 1950); 3. socio-economic data on occupation and literacy drawn from censuses, population lists and parish registers. 

We used height data for the military conscripts enlisted in the 14 villages mentioned in Section 2 between 1835 and 1977. Of these data, 91.8% were obtained from the records kept in the municipal archives of each village. To complete the sample, we requested a copy of the available conscriptions in the Historical Military Archive of Guadalajara. From this archive, we were able to identify 206 new individuals. The final sample included a total of 2510 men who can be followed throughout their lives (see Table 2 for their distribution by birth cohort). Of these 2510 conscripts, we can identify all their fathers; however, we only have height information for 521 of these fathers (due to paucity of data for the nineteenth century and the first decades of the twentieth century). The information on paternal height (intergenerational transmission) enables us to analyse the pioneers of the transition in relation to their height and that of their children.

**Figure A1:**
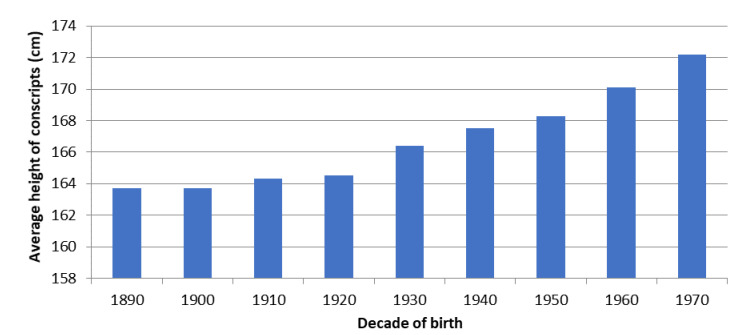
Evolution of the average height of conscripts in the study area according to decade of birth, 1890–1979 (Source: AMHDB. *n* = 2510 men). Note: See Appendix A, Table A4 for the number of observations, standard deviation and 95% confidence intervals.

During the period of study, the conscription age varied over time. During the period 1856–1885, the age of military conscription was 20 years old; between 1885 and 1899, it was 19 years; between 1901 and 1905, it was 20; and between 1907–1939, it was 21 years old. Thus, we have standardised the average height to the age of 21 years. To do this, we have used the same strategy as Ayuda and Puche-Gil [41] based on calculating the 50th percentile of the three age groups (19, 20 and 21 years), adding 1.2 cm to the height of 19-year-olds and 0.4 cm to the 20-year-olds. Our results are similar to those obtained for other Spanish regions [41,74]. The distribution of the height data is close to normal for the whole period. We have tested the null hypothesis of normality of average height and cannot reject the null hypothesis at a significance level of 5%. Improvements in living standards allowed for a steady increase in average height throughout the study period (see Figure A1 in the Appendix A for the distribution by decade).

The demographic event analysis is based on the complete church registers of these 14 villages. These records provide high-quality information on all baptisms, marriages and deaths that occurred between the sixteenth century and 1950, although the starting date varies by location. For more details about the ‘Alfamén and Middle Huerva Database’, see [75,76,77]. To obtain similar data for the period after 1950, 1074 interviews were conducted with relatives of the individuals analysed. The interviewees were asked about the dates of demographic events, occupation, and education. The mortality data were completed with information obtained from public sources linked to the cemeteries in each locality. The database was built following the family reconstitution method devised by Fleury and Henry [52]. It includes all individuals who were born and baptised in the reference parishes or who migrated to them and were registered there in connection with one of the previously described events. This dataset contains information on approximately 125,000 individuals, including name, sex, place and date of birth, parents’ names and date of death, among other details, enabling us to reconstitute the life history of these individuals and their families. Great effort was required in order to link all parish data, height data and complementary sources due to the limitations of the data availability in most of the villages.

The occupation of the individuals analysed and their fathers were taken from population lists (1857 and 1860), electoral censuses (1890, 1894, 1900, 1910, 1920, 1930, 1934, 1945, 1951 and 1955), and parish registers. Information on profession, and height was linked to population records for each individual. 

### 2.3. Methods

We have used bivariate analysis to determine the height-group to which the pioneers of the fertility transition belonged, how fertility control affected the average height of the children and whether the number of family members living in the same village affected the possibilities of controlling fertility and the biological well-being of the children. The results of these analyses reveal the origins of the fertility transition and whether there was a relationship between height and the different stages of the transition. Furthermore, in the Appendix, the main analyses are replicated by dividing the individuals analysed according to their socio-economic status for the two main groups in the study area: agricultural labourers (1,1,1,30) and husbandmen (1,1,1,3). Data regarding occupation are coded in PSTI [78]. This system is based on a PST occupational coding scheme [79] but is internationally adapted. This approach allows us to conduct a comparative analysis of the profile of the pioneers of the fertility transition and their children, differentiating between individuals who controlled their fertility (the next subsection explains how we have identified them) and those who maintained traditional demographic behaviours.

This study uses descriptive statistics and statistical tests, such as confidence intervals, to approach the main question of the article on the relationship between fertility control and height. Therefore, most figures and tables have, as the main variable studied, the average height of a given group of individuals according to the birth cohort and their individual characteristics. Other variables studied are the average number of children and the average age at a given event. This simple methodology allows us to make a first approximation to the main question taking into account the small sample size available and the available variables. However, this sample size (together with the variability of heights between human beings due to genetic and other factors) is also sometimes a problem. 

If we take into account the 95% confidence intervals of the control families and the rest, most results (with means) are not statistically significant. However, a *t*-test approximation allowed us to validate that our approximation is not erroneous overall, although unfortunately (possibly due to the sample size in some periods), the *p*-values are higher than 0.05 in the early decades, so the results for these decades should be taken with extra caution. In any case, with this methodology, we mainly want to study the temporal evolution (by decades) and trends of some groups with others. In our case, the trends are clear and consistent with what would be expected from the evolution of height and the fertility transition. 

In addition, we performed six multivariate linear regression models OLS with heteroskedasticity-robust estimation linking height and fertility control in which the dependent variable is the height of the individual (in millimetres). The objective is to confirm whether there is a relationship between height and parental fertility control, controlling for various socio-economic, health and family factors. Model (1) includes fertility control, locality of residence and decade of birth as independent variables. Model (3) builds on model (1) and also includes parental socio-economic status and individual literacy. Model (4) also includes the claims made by the individual not to participate in compulsory military service. Model (6) includes all of the above variables plus the number of living siblings (given that in all cases, the parents were over 49 years of age). Model (2) is similar to model (3) but without controlling for decade of birth and village. In the same way, model (5) is similar to model (6) but without controlling for decade of birth and village. Model (6), which is the most complete, can be expressed as follows:

(4) HEIGHT_i_ = β_1_ × CONTROL_i_ + β_2_ × SES_i_ + β_3_ × LITERACY_i_ + β_4_ × APPEAL_i_ + β_5_ × FAMILY_i_ + β_6_ × VILLAGE_i_ + β_7_ × DECADE_i_ + ε

where HEIGHT is the height in millimetres of an individual i, CONTROL is a discrete variable for if the individual’s parents stopped having children before the age of 36, SES is the socio-economic status of the parents (as a proxy for the level of family economic well-being), LITERACY is the literacy of the individual (as a proxy for the parent’s investment in their children, although we should bear in mind that during much of the study period, all children were literate), APPEAL is the claims made to evade military service and which were accepted by the competent authority, differentiating between physical problems and social problems linked to the existence of siblings in the military and family poverty. FAMILY is the family size of living siblings who exceeded five years of age, VILLAGE is a control variable on the locality of residence within the study area, and DECADE is the decade of birth of the individual. Decade of birth captures many evolving circumstances of biological, ecological, and cultural nature that were important for the general trend in biological well-being and heights. As we will see in the results section, the results of the regression models clearly confirm (at 99% significance) that this relationship existed and was strong, and our previous results are not spurious.

### 2.4. Fertility Control Using Stopping

Stopping was the most common fertility control strategy during the fertility transition in Western countries, and the strategy most clearly associated with fertility control [1,19,20,21,23,24,80,81,82,83]. Stopping is when families voluntarily stop having children at an unusually young age. Undoubtedly, it was the most widespread strategy in our study area [17]. Before the fertility transition, approximately 10% of households stopped having children at an unusually early age (possibly due to health problems), whereas more than 50% of households made use of this strategy during the later stages of the transition, either as the sole strategy or in combination with others.

Because of its characteristic of abruptly detaining fertility, stopping is easier to identify than other fertility control strategies such as spacing [61]. Moreover, although there is a margin of error due to families who stopped having children at an early age because of fecundity problems, the reality is that as the transition progressed, the vast majority of families who stopped having children at an early age did so voluntarily. Therefore, we will consider families that stopped having children at an early age as families that voluntarily controlled their fertility. Furthermore, we will include in this group (families that used stopping) those in which the woman had her last child before the age of 36. Thus, if the mean age of women at last child in the pre-transitional period was slightly above 39 years (in 1800–1860, the mean age at last child was 39.1 years). Women who used the stopping strategy were childless for more than three fertile years (which is a gap above the mean birth interval). To establish this threshold, we followed the criterion established by Alberto Sanz and Fernando González [23], which allows us to analyse the evolution over time of the individuals who used stopping. They established that fertility control behaviour could be established when a family stops having children at least three years before the mean age of the last child is in pre-transitional stages. The marriages included both spouses over 49 years of age and, therefore, should have completed their childbearing cycle, and we have no record of them having separated or divorced. However, not all those who stopped having children before the age of 36 did so by a consensual decision of the spouses. There are other reasons, such as secondary infertility or marital discord, that may also have affected the cessation of fertility. In short, the methodology used is arbitrary, but it allows us to identify most of the families that used stopping. Furthermore, depending on the age at marriage and birth intervals, family size varies. However, as we will see below, there is a relationship between stopping fertility before the age of 36 and smaller average family size.

Therefore, from this point onwards, and for the purpose of this study, we will use the imperfect criterion that families who stopped having children before the woman reached the age of 36 (and both parents were alive at the age of 49) were the families that voluntarily controlled their fertility.

## 3. Results

First, we will briefly examine the period before the fertility transition and compare it with the transitional period. This requires an in-depth analysis of the intergenerational transmission of height between fathers and sons periods and a comparison of whether there was any modification during the fertility transition. Table 3 displays, by tertiles (tall, medium and short), fathers and sons in relation to the rest of men born in the same decade in the period prior to the fertility transition. This transition started in the study area with children born in the first decade of the twentieth-century corresponding to fathers born in the last decades of the nineteenth century [17]. The aim of Table 3 is to test whether the children were in the same height tertile as the parents, which would confirm that a transmission of height from parents to children was taking place. This behaviour could be conditioned by genetics or by the family’s socio-economic situation [34,35,38,39,40,41]. We analysed the birth cohorts (of the fathers) of 1835–1889. A limitation of this table is that it only takes into account the father, without knowing the height and socio-economic status of the maternal family. The results in Table 3 show that, before the fertility transition (and in its early stages), children had more than a 50% chance of belonging to the same tertile as their fathers. Thus, children and parents tended to have a similar level of biological well-being. Before the fertility transition, there was a traditional relationship linking parents with low biological well-being with children with low biological well-being, and vice versa.

In Table 4 we performed the same analysis, but for the birth cohorts (of fathers) of the period 1890–1919, during the first stages of the fertility transition. The results change greatly, as the sons seem to have improved their biological well-being. A higher proportion of sons grew to reach higher tertiles than their fathers (relative to Table 3). In fact, maintaining the same status as the father did not reach 50% of the cases. This especially affected the children of short fathers in a positive way. Thus, two key observations may be drawn from Table 3 and Table 4. The first is, as we previously confirmed, other factors than just genetics alone explain the tertile and biological well-being each individual is placed in. Second, at least in the study area, the fertility transition served as a mechanism to break traditional circles and allow the lowest status individuals to improve the biological well-being of their children. How these vicious traditional circles were broken, by whom and what consequence it had on offspring will be explored in this section.

### 3.1. The Pioneers of the Fertility Transition

**Figure 2:**
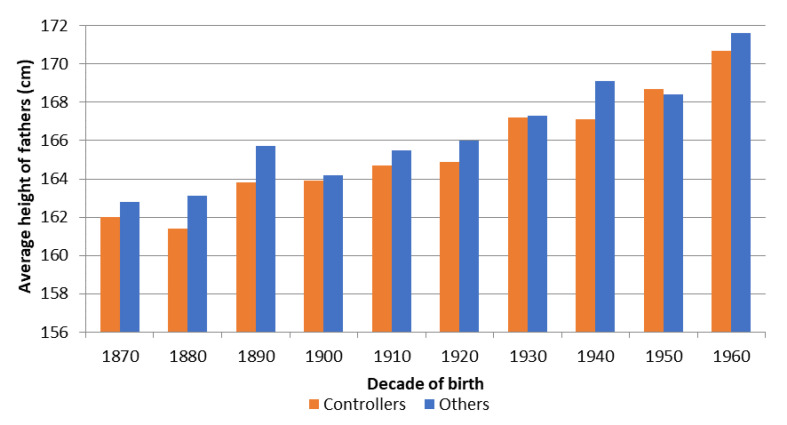
Average height of fathers of complete families, according to whether they controlled their fertility by stopping or not, birth cohorts 1870–1969 (Source: AMHDB. *n* = 1102 men). Note: See Table 5 for the number of observations, standard deviation and 95% confidence intervals.

**Figure A2:**
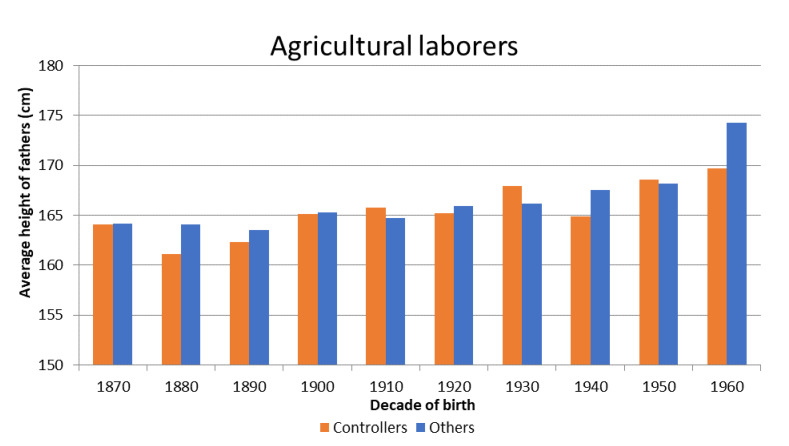
Average height of agricultural labourers’ fathers, according to whether or not they controlled their fertility by stopping, birth cohorts 1870–1969 (Source: AMHDB. *n* = 2510 men). Note: See Appendix A, Table A5 for the number of observations, standard deviation and 95% confidence intervals.

**Figure A3:**
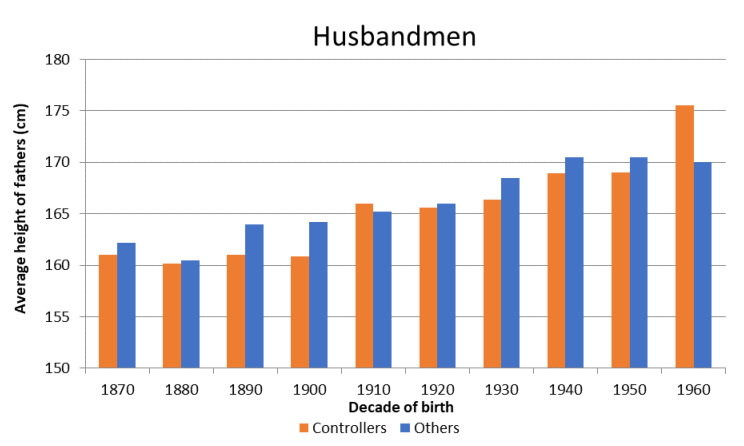
Average height of husbandmen’ fathers, according to whether or not they controlled their fertility by stopping, birth cohorts 1870–1969 (Source: AMHDB. *n* = 2510 men). Note: See Appendix A, Table A6 for the number of observations, standard deviation and 95% confidence intervals.

First, we will analyse who the pioneers in controlling fertility by using the stopping strategy were in relation to their biological well-being. In this way, we can determine whether they were the local elite individuals (who we would expect to be the conscripts with the tallest average stature). In Figure 2, we observe that, in general, throughout the fertility transition, parents who controlled their fertility by stopping were on average shorter than the rest of individuals (those that we cannot identify as fertility controllers by stopping). In fact, the average difference was greater than a centimetre, being especially large in the early stages where it was almost two centimetres among men born in the 1880s and 1890s. When we analyse the same patterns by socio-economic status for the two main socio-economic groups in the study area: agricultural labourers and husbandmen (see Figure A2 and Figure A3 in the Appendix), again the patterns are repeated, with the gap being especially large (with a mean of more than two centimetres) in the case of husbandmen. Unfortunately, as can be seen in Table 5, the confidence intervals do not guarantee statistical significance at 95%. Therefore, we have some trends that are consistent with the rest of the Figures and Tables and that help us to further investigate the relationship between height and fertility control, but we should take the results with caution.

These results seem to suggest that it was mostly individuals from poorer households (and thus those who had received poorer nutrition and/or care, which was reflected in their biological well-being) who had more incentives to control their fertility. As we will discuss in the next section, these results seem to contradict the perception that it was local elites who were the pioneers of the fertility transition. In fact, in Table A1 of the Appendix, we can see, once again, that shorter individuals controlled their fertility in a clearly higher proportion than the rest of the individuals and that there was a negative relationship between the percentage of controllers and height. 

Stopping proved to be a very useful strategy for reducing marital fertility. Table 6, compares the mean total number of living children of individuals who controlled their fertility by stopping with the rest of individuals by decade. The results clearly show that individuals who used stopping drastically reduced their fertility to almost half as many live children as those who did not exercise any (identified) control in the early stages. In the 1870s and 1880s, while the others had more than four children on average, the controllers had barely more than 2.5 children on average. By comparing the pre-transition offspring size with the values calculated for controllers and others, we can conclude that the strategy used is very useful, up to those born in the 1890s. From that moment on, even among the others, fertility control is taking place, which we cannot identify with the selected strategy. In fact, in some cases, there have been controlling families (included in the ‘others’ group) who, although they were controlling their fertility, failed to stop it completely and had a child (possibly unwanted) beyond the age of 36.

Moreover, we know that the differences in the decision to control fertility were not due to different ages of entry into marriage since, as Table A2 in the Appendix A shows, all stature groups have similar ages of entry into first marriage. Possibly, in the study area, families with fewer resources (and therefore lower levels of biological well-being) had the greatest incentive to control their fertility due to the pressure exerted by the increase in family size on the family budget [24]. Additionally, there could also be a direct relationship between controlling fertility and the female opportunities in the job market:

*Demographic factors such as the fertility rate, infant mortality or the type of household (in particular, the ratio between adult women and dependent individuals within the household), decisively condition the availability of women for paid employment: the number of hours, the times of their life cycle and the type of employment in which they can occupy. And it also explains the relationship between the massive diffusion of contraceptive means in the second half of the 20th century and the fall in fertility and the continued increase in the female activity rate in all developed countries.*

[84]

Without seeking to carry out an exhaustive analysis, we can refer to different authors who have conducted research on the issue for the period of study. For the nineteenth century, studies have been carried out by Camps [85], Borderías [86] and López-Antón [87] for Catalonia; Muñoz-Abeledo [88], and, Muñoz-Abeledo, Taboada and Verdugo [89] for Galicia; or Campos-Luque [90] for Andalusia. The results differ from one another, mainly depending on the age chosen to consider dependent children, the type of municipality (rural or urban), the place of work of women (within or outside the home) and the existing job opportunities for women in each area of study. Currently, the question remains as to which were the most influential variables for determining the supply of female labour, those on the supply side or those on the demand side. In any case, the decision to control fertility not only affected the number of people in the household between whom the available budget had to be divided but probably allowed women who controlled their fertility to have better access to the labour market as they had fewer dependent children in the household, thus increasing the family budget.

### 3.2. The Effect of the Fertility Transition on the Biological Well-Being of Offspring

**Figure 3:**
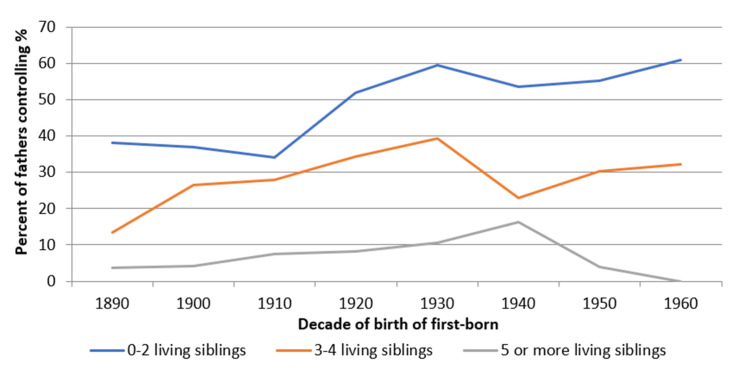
Percentage of individuals who controlled their fertility by stopping, by number of children, classified by year of birth of first-born, 1890–1960 (Source: AMHDB. *n* = 2510 boys).

**Figure A4:**
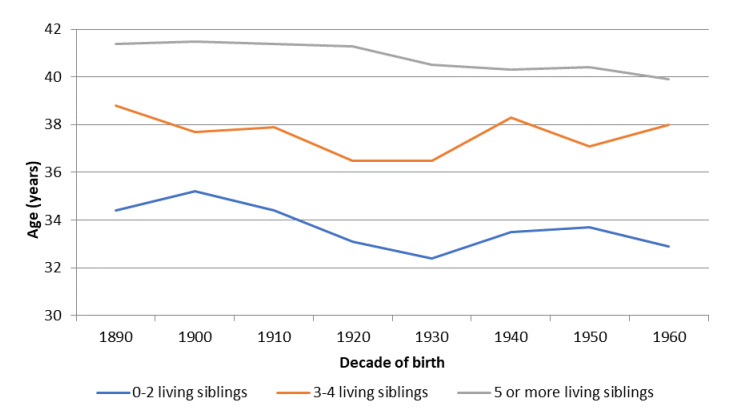
Mean age of mothers at last child in complete families as a function of the number of living children, birth cohorts 1890–1969 (Source: AMHDB. *n* = 1989 boys).

This subsection addresses the relationship between height and fertility control from the perspective of children and the influence that the fertility decisions had on them. As we can observe in Figure 3, fertility control by stopping had an effect on the reduction of the family size. Thus, while from the 1920s onwards, more than 50% of the families that had two living children or less (of final offspring) controlled their fertility by stopping, only a third of those that had 3–4 living children did so, and less than 10% of those that had five or more children. That is, the family size was strongly conditioned by the decision to control fertility. Similarly, the ‘age at last child’ (Appendix, Figure A4) was also linked to the decision to control fertility and the number of living children, where families with fewer living children visibly reduced their age at last-child voluntarily.

**Figure 4:**
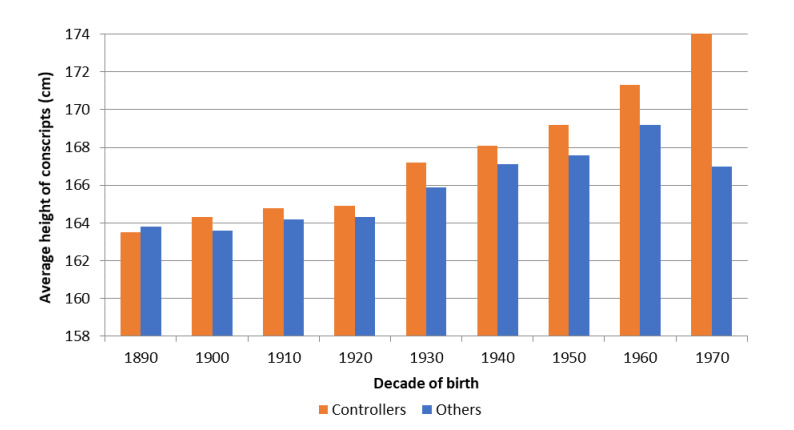
Mean height of males at 21 years old in complete families according to whether or not their parents controlled their fertility by stopping, individuals born between 1890 and 1979 (Source: AMHDB. *n* = 2510 boys). Note: See Table 7 for the number of observations, standard deviation and 95% confidence intervals.

**Figure A5:**
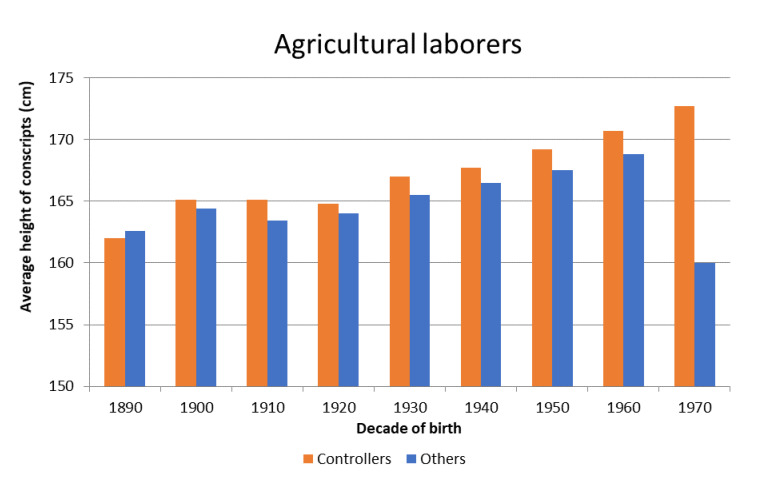
Mean height of children of agricultural labourers, according to whether or not they controlled their fertility by stopping, birth cohorts 1890–1979 (Source: AMHDB. *n* = 1312 boys). Note: See Appendix A, Table A7 for the number of observations, standard deviation and 95% confidence intervals.

**Figure A6:**
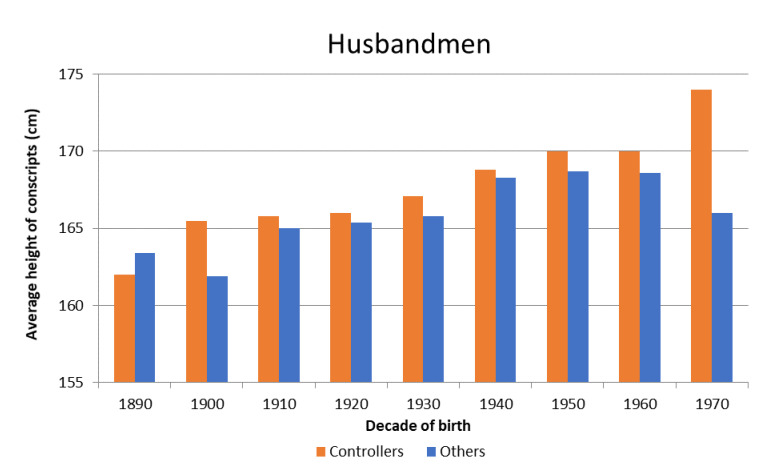
Mean height of children of husbandmen, according to whether or not they controlled their fertility by stopping, birth cohorts 1890–1979 (Source: AMHDB. *n* = 476 boys). Note: See Appendix A, Table A8 for the number of observations, standard deviation and 95% confidence intervals.

What effect did stopping have on the biological well-being of children? To answer this question, Figure 4 shows the average height attained by children at age 21 as a function of whether parents controlled their fertility. The results reveal that children of controlling parents were, on average, taller than the rest of the children from the beginning of the fertility transition. In addition, the results also show that the gap in height increased as the transition progressed, from an average difference of less than one centimetre between those born in 1900–1929 to differences of close to two centimetres between the birth cohorts 1950–1969. In the Appendix A (Figure A5 and Figure A6), we have performed a similar analysis according to the socio-economic status of the father. Both Figures confirm the results obtained in Figure 4; children of fathers who controlled their fertility were on average taller than the rest. The gain in height was particularly large among the children of agricultural labourers (with increases of close to two centimetres), possibly because of the greater budgetary constraints faced by poorer socio-economic groups. These results seem to show that fertility control resulted in an improvement in per capita household income, leading to an improvement in children’s diet and height. Whether or not these improvements in biological well-being were the consequence of parents’ willingness to reduce their fertility and invest more in their children’s well-being is an interesting question that has been taken into account in the discussion section. Again, the results in Table 7 should be taken with caution. Figure 4 shows a trend consistent with the results obtained so far (with our small sample), but we need to confirm this relationship through statistical regressions (as we will do later).

**Figure 5:**
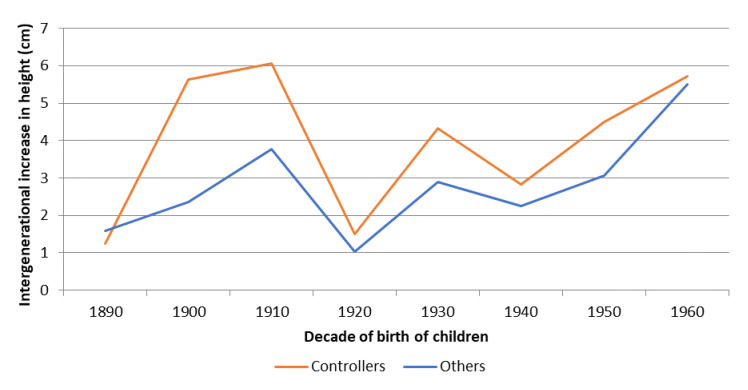
Average intergenerational increase in height (in cm) between fathers and children as a function of whether or not parents controlled their fertility by stopping, children born between 1890 and 1969 (Source: AMHDB. *n* = 459 gaps).

The results are particularly interesting if we take into account that short individuals could be the pioneers of the fertility transition in the study area. As we have shown, before the fertility transition, parents of the lowest tertile of biological well-being mostly passed this condition on to their children. However, once the transition began, parents with a low level of biological well-being were those who controlled their fertility and started having children of above-average stature. That is, the fertility transition served as a mechanism to break the cycle that had condemned parents and children to remain at similar levels of biological well-being from one generation to the next. The children of controllers achieved mean heights above those obtained by their peers of other parents, regardless of whether socio-economic status is controlled for. Figure 5, shows the mean intergenerational improvement in height (from fathers to sons) as a function of whether or not parents controlled their fertility by stopping. The results are clear and consistent with those obtained up to this point. The sons of fertility-controlling parents increased their height by 50% more than the sons of other parents. This intergenerational increase was especially important in the early stages of the fertility transition; while the children of controlling families increased their height with respect to their fathers by up to six centimetres in the 1900s and 1910s, the children of other families only had a gap of between 2.5 and 4 centimetres. As the fertility transition progressed and the gains accumulated, the gap tended to decrease during the following decades to 20–25%. In the last decade we analysed (the 1960s), significant improvements in terms of standards of living and economic status were occurring in Spain, and there was almost an equalisation between the two groups.

The results in Figure 5 could be conditioned by the evolution of the political situation as well as by the fertility control at household level. Thus, we observe that between those born in the 1900s and 1910s, there were important differences in intergenerational height gain depending on whether parents controlled their fertility. However, these differences narrowed for both controlling and other families in the following decades. This could be the consequence of the effects of the Civil War and autarky on the biological well-being of children (in comparison to their parents). Their visible increases in stature due to the economic and health improvement could have been reduced by the large negative shock of the Spanish Civil War and the subsequent food shortages.

To ensure that there was a relationship between fertility control and height, we have developed six multivariate (OLS) linear regression models with heteroskedasticity-robustness estimations controlling for various socio-economic, health and family factors (see Table 8). The dependent variable in all models is height, so we are performing an analysis of the determinants of height. The results confirm with high significance the relationship between parents controlling their fecundity by stopping and a greater height of their children. In other words, what has been shown throughout this article with bivariate analysis is confirmed with multivariate analysis. When we have more complete models capable of controlling for various factors, the results become significant (even in almost all cases at 99%). Similarly, the hypothesis that the children of individuals from poorer socio-economic groups were shorter than the children of parents from other groups, and vice versa, is confirmed for the study area. For the children of upper-class parents, we also found a greater height being significant at 90%. We can also observe that the decade of birth, as expected, plays a major role in the determinants of height. However, the relationship between height and fertility control is highly significant, with results above 10 mm in all cases (including models (2) and (5) where birth decade and village are not controlled for). While other factors, such as literacy or family size, are much more affected by the decade of birth. Finally, it should be noted that physical health problems cited as a reason for not going to military service were also a factor negatively correlated with height.

### 3.3. Family Group

This last subsection examines whether the number of immediate living family members (grandparents, uncles and aunts of the children born) residing in the same village as the children (not necessarily in the same house) had any influence on the likelihood of their parents reducing their fertility using the stopping strategy during the fertility transition. Several studies have demonstrated the importance of kin, and most especially grandmothers, in children’s well-being [91,92,93]. The results in Table 9, in which we have classified the number of aunts, uncles and grandparents residing in the same village at the time of the child’s birth (0–2 persons, 3–5 and 6 or more relatives), seem to confirm the existence of a positive relationship between the number of relatives and the height reached by the conscript at age 21. The greater the number of adult relatives that can help raise children, the taller the height. For the whole period, individuals with two or fewer relatives were, on average, 1.6 cm shorter than those with six or more relatives. Those with three to five relatives were 1.1 cm shorter. These results could be explained by the positive effect of having very close relatives in the same locality (cooperative breeding) and its impact on the care and nutrition of the new generations. Family members were able to provide financial and nutritional coverage for children during the early stages of life. In any case, we cannot state categorically that this is the explanation for the results obtained—only the existence of this relationship.

However, when we studied whether the number of relatives had any impact on the decision to control, as we can see in Appendix A Table A3, we found no clear pattern. The variations occur almost randomly. The results seem to indicate that the number of family members had a positive effect on parenting but did not have a considerable effect on the development of the fertility transition and the desire to control family size.

## 4. Discussion

The results obtained in this study help us understand the effect of fertility control on the biological well-being of those born during the twentieth century in the study area. Research carried out for different Western countries, and other large areas tend to confirm that it was families belonging to the urban elite classes who began to control their fertility [24,25]. Moreover, the areas that were better communicated for the arrival and diffusion of new ideas seem to have begun their transition earlier than other nearby and similar but less well-communicated areas [94]. However, in the area of study, at first sight, the results differ from the studies cited above. From the beginning of the fertility transition, shorter individuals (families with poorer early life conditions) were those who controlled their fertility by stopping. The local elites, with a higher nutritional status (and probably health status), do not seem to have preceded the rest of the families in fertility control. In other words, there was no downward transition in the social pyramid. However, the results obtained are not incompatible with the literature cited above. We have no detailed knowledge of how the ideas linked to the desire to control fertility and control strategies were transmitted from the elites to the working classes. It is possible that, in the case of villages, it was not necessary for local farmer elites (usually conservative) to take the initiative. Individuals were able to receive and embrace new ideas through regional elites or other urban groups through commercial contacts.

Classical fertility theory suggests that it was the increase in family size and its pressure on the family budget that led families to control their fertility [24,27]. Due to the fall in infant mortality after the second half of the nineteenth century, families increased their average size in a rural context of near-subsistence living standards [95]. In this context, it is possible that poorer families had more incentives to control their fertility by making use of the new ideas of fertility control and, especially, of the most popular strategy: stopping. Families that reduced their fertility were better able to nurture their children and thus increase their biological well-being. Therefore, the fact that a higher proportion of people with low-level biological well-being were the pioneers of the fertility transition could be due to the need to balance family size and budget, at a time when food and consumption patterns were changing towards more expensive products (such as meat) [96]. It is possible that fertility control can reduce pressure on the family budget while also allowing an increase in the budget derived from the women’s labour supply.

The results obtained here also show that before the fertility transition, individuals with a lower level of biological well-being tended to mostly have children with a low level of biological well-being, and vice versa. Therefore, a vicious circle ensued in which levels of biological well-being were transmitted from parents to children (as we have seen in Table 3 and Table 4). However, in the study area, the fertility transition involved a process of social, economic and biological well-being transformation. The results have confirmed that such intergenerational conditioning ceased to be the norm after the fertility transition. In fact, the children of men who controlled their fertility by stopping were, on average, taller than other children of the same generation. Moreover, Figure 5 confirms that children of individuals who controlled their fertility were taller than their parents by up to 50%, in comparison with other families. Social mobility was no longer the main mechanism for increasing biological well-being. The decision to control fertility was an alternative mechanism available to all individuals. Therefore, the demographic transition goes beyond being a demographic process to becoming a process of economic and social transformation [5]. Consequently, the resource dilution hypothesis would be applied to benefit socio-economic change [66,67]. A large proportion of poor families would have voluntarily reduced their family size and, in so doing, would have increased biological well-being for their offspring. In general, competition for food among poor families is correlated with low nutritional status and low adult height and is an incentive for fertility control; smaller family size implies better nutrition for children and, therefore, higher adult height. The transition process was based on changes in the behaviour of the families who were the architects of the improvements in the biological well-being of the new generations.

Another interesting question arising from the results is why the children of fertility-controlling parents were taller than the rest. The obvious answer, given family budget constraints, is that families with fewer children were able to invest more in their offspring. Hatton and Martin [56] also found similar results for the British case. However, the remaining question is whether parents decided to control their fertility with the intention of investing more in their living children (trading quantity of children for quality of children) as proposed by Becker [68]. On the one hand, research in several Western countries suggests that tall individuals had higher wages [97,98] and more success in the marriage market [99,100,101]. Therefore, the parents most negatively affected by this discrimination may have been those most interested in their children breaking away from vicious historical circles. The reduction in the number of living children and, therefore, the higher per capita budget in the family may be the cause of the increase in stature, without it necessarily being an effect sought by the parents, but rather a consequence. This situation would, therefore, represent the resource dilution hypothesis without human agency. Unfortunately, there is little data to test human agency. At the beginning of the twentieth century, the area of study reached full literacy, and access to secondary and post-secondary education required a costly move to other larger localities so that very few individuals had the possibility to increase their educational level. Due to these limitations, a good proxy for investment in children, namely educational attainment [102,103], is not a useful variable for this research. Neither can conclusions be reached based solely on occupation or offspring mortality. This aspect remains one of the fundamental questions for future research. What is certain, however, is that the offspring of controlling parents enjoyed significant improvements in stature in relation to their parents and peers. The results so far seem to confirm that fertility control may have served in rural Spain to improve the biological well-being of existing offspring.

Throughout this article, we have focused on the explanations centred on the differential behaviour of fathers. However, there are other complementary biological factors that we have not discussed that could be linked to the tendency of increased height, even during the strong negative shock of the Spanish Civil War. The most prominent of these is the exposure of individuals to pathogens [104]. Sanitary, health, scientific and economic improvements could have led to healthier environments, with less exposure to harmful bacteria and viruses. This could have led to an increase in the biological well-being of children born as parents invested less (biologically) in fighting disease. In any case, these changes in morbidity are unlikely to explain the differences in height between the children of controlling parents and the rest, beyond the fact that a fuller house with fewer square metres per inhabitant could influence the transmission of diseases among members of larger families.

### Limitations

As previously explained, the data used in this study have several limitations. On the one hand, the small sample size and the fact that they correspond to a rural agricultural area composed of 14 villages may pre-condition the results. We cannot be sure that the behaviour in these villages does not differ from that of urban areas or other rural areas with different socio-economic distributions or historical characteristics. On the other hand, we are only analysing anthropometric data for men, so any gender discrimination in relation to the investment in biological well-being could bias our results. In any case, Marco-Gracia and Beltrán Tapia [59] show that gender discrimination tended to disappear in rural Aragón during the first decades of the twentieth century. In addition, there are other limitations associated with the data, such as our inability to confirm that these patterns were replicated in other areas (although the selected area has a similar socio-economic and spatial structure to most of dry, inland Spain).

Additionally, the small sample size (and data availability) conditioned our decisions on methodology, analyses performed and depth achieved. In fact, as we mentioned when discussing methodology, and would like to point out again, the descriptive results in several cases have problems of non-significance due to the small sample size (see Table 5 and Table 7, Table A4, Table A5, Table A6, Table A7 and Table A8). Unfortunately, with descriptive statistics alone, we cannot confirm our results with 95% statistical significance (although the *t*-tests have reported positive results for the periods with more observations). However, the trends in the results are clear and permanent, which was our objective in using this methodology in the study. Therefore, further studies with larger sample sizes are needed to confirm or refute our tentative findings.

To complement the analyses with descriptive statistics (and the associated problems described), we developed an analysis with six multivariate (OLS) linear regression models that, in all cases, confirm a strong correlation between fertility control and height in the study area. Individuals who controlled for fertility had taller children than their contemporaries from families in the area who were not included in the fertility control group. However, more research is needed to further investigate this relationship.

## 5. Conclusions

This paper analyses the fertility transition at the individual level in a small Spanish rural area using height data (proxy of biological well-being) at age 21. The objective is to determine the pioneers of the fertility transition and the effect that controlling fertility had on the biological well-being of the next generation. In other words, rethinking the fertility transition in relation to height (as an indicator of living standards).

Traditionally, prior to the fertility transition, there was a positive relationship between height and socio-economic status [34,35,38,39,40,41], with individuals of low socio-economic status having a shorter-than-average height. In a context of high fertility and mortality, levels of biological well-being and socio-economic status were transmitted from parents to children. Parents in the shortest height tertile (relative to their peers) tended to have children who were in the shortest tertile (relative to their own peers). However, this relationship was broken during the fertility transition as short parents were more likely to control their fertility by stopping (the fertility control strategy associated with the fertility transition). Possibly, family budget constraints were incentive enough for taking the decision to reduce marital fertility.

Stopping fertility had positive effects on the next generation. The children of families that controlled their fertility were, on average taller than the children of the rest of the children. In fact, the intergenerational increase in height during this period of economic modernisation was 50% higher among parents who controlled their fertility. That is, fertility control was strongly beneficial at the level of biological well-being for children, especially for the children of individuals from the lowest socio-economic groups. In the area of study, the fertility transition could have constituted a way to break the traditional poverty circles.

## Figures and Tables

**Table 1:** Population of the villages in the sample 1860–1981.

	Population			
	1860	1900	1940	1981
Alfamén	604	639	1347	1283
Aylés	45	26	47	0
Botorrita	294	350	557	382
Codos	1232	1195	938	355
Cosuenda	1451	1270	929	482
Jaulín	390	348	528	334
Longares	1120	1329	1385	959
Mezalocha	544	482	660	357
Mozota	292	372	404	158
Muel	1223	1206	1605	1330
Torrecilla de Valmadrid	164	77	94	32
Tosos	682	865	801	297
Valmadrid	203	210	219	89
Villanueva de Huerva	690	970	1158	771
TOTAL	7926	8196	10,672	6829

Source: Spanish Statistical Institute (www.ine.es/intercensal/ (accessed on 5 August 2021)).

**Table 2:** Number of conscript observations by decade of birth.

Birth Decade	Number of Obs.	Birth Decade	Number of Obs.
1890s	113	1940s	386
1900s	312	1950s	251
1910s	330	1960s	113
1920s	561	1970s	11
1930s	463	TOTAL	2510

Source: Alfamén and Middle Huerva Database (AMHDB).

**Table 3:** Correspondence between height categories of fathers and sons before the fertility transition (fathers born in 1835–1889), in percentage.

		Sons		
		Short	Medium	Tall
FATHERS	Short	59	26	15
	Medium	32	50	18
	Tall	9	24	67

Source: AMHDB. *n* = 146. Note: Fathers and sons have been distributed by tertiles of height according to their decade of birth. In 1830s, short are <155.0 cm, tall >156.8; in 1840s short are <161.0 cm, tall >164.4; in 1850s short are <164.0 cm, tall >166.9; in 1860s short are <161.0 cm, tall >164.9; in 1870s short are <162.0 cm, tall >165.9; in 1880s short are <161.0 cm, tall >165.9; in 1890s short are <162.5 cm, tall >167.9; in 1900s short are <161.0 cm, tall >166.9; in 1910s short are <162.5 cm, tall >167.0; in 1920s short are <163.1 cm, tall >168.1; in 1930s short are <164.8 cm, tall >170.4; in 1940s short are <166.0 cm, tall >170.0; in 1950s short are <168.0 cm, tall >170.9.

**Table 4:** Correspondence between height categories of fathers and sons during the first stage of the fertility transition (fathers born in 1890–1919), in percentage.

		Sons		
		Short	Medium	Tall
FATHERS	Short	45	45	10
	Medium	29	26	45
	Tall	26	29	45

Source: AMHDB. *n* = 168. Note: Fathers and sons have been distributed by tertiles of height according to their decade of birth (see note in Table 3).

**Table 5:** Average height, standard deviation and 95% confidence intervals of fathers of complete families, according to whether they controlled fertility by stopping or not, birth cohorts 1870–1969. These data correspond with Figure 2.

	Controllers					Others				
Decade	Observation	Average	Standard Deviation	95% Confidence Interval		Observation	Average	Standard Deviation	95% Confidence Interval	
1870	8	162.0	1.3	159.1	165.1	25	162.8	0.9	160.9	164.7
1880	15	161.4	1.0	159.2	163.7	25	163.1	1.2	160.6	165.6
1890	29	163.8	1.1	162.8	164.9	24	165.7	1.0	163.7	167.8
1900	107	163.9	0.5	162.8	164.9	70	164.2	0.7	162.8	165.7
1910	62	164.7	0.7	163.3	166.2	71	165.5	0.8	164.0	167.0
1920	153	164.9	0.4	164.2	165.7	106	166.0	0.6	164.8	167.2
1930	98	167.2	0.6	166.1	168.3	70	167.3	0.6	166.1	168.6
1940	61	167.1	0.6	165.9	168.4	54	169.1	0.7	167.7	170.6
1950	48	168.7	0.7	167.4	170.1	39	168.4	0.9	166.6	170.2
1960	24	170.7	1.2	168.2	173.3	13	171.6	1.3	168.9	174.4

Source: AMHDB.

**Table 6:** Average number of living children (5 years or more) of parents who did and did not control their fertility by decade of birth, birth cohorts 1870–1939.

	1820s–1860s	1870s	1880s	1890s	1900s	1910s	1920s	1930s
Whole group	3.9	3.8	3.6	2.5	2.1	2.2	1.7	1.8
Controllers	-	2.7	2.6	1.7	1.7	1.6	1.5	1.4
Others	-	4.1	4.0	3.3	2.4	2.6	2.0	2.1

Source: AMHDB. *n* = 1102 men. Note: Only parents who have had at least one child are considered. For the period before the fertility transition (1820s–1860s), we do not differentiate between controllers and others.

**Table 7:** Average height, standard deviation and 95% confidence intervals of males at 21 years old in complete families, according to whether or not their parents controlled their fertility by stopping, birth cohorts 1890–1979. These data correspond with Figure 4.

	Controllers					Others				
Decade	Observation	Average	Standard Deviation	95% Confidence Interval		Observation	Average	Standard Deviation	95% Confidence Interval	
1890	15	163.5	1.4	160.5	166.5	98	163.8	0.6	162.6	164.9
1900	50	164.3	0.8	162.7	166.0	262	163.6	0.4	162.9	164.4
1910	55	164.8	0.8	163.2	166.5	245	164.2	0.4	163.4	165.1
1920	164	164.9	0.5	163.9	165.9	397	164.3	0.3	163.8	165.0
1930	195	167.2	0.4	166.4	168.0	268	165.9	0.4	165.2	166.6
1940	156	168.1	0.5	167.1	169.2	230	167.1	0.4	166.4	167.9
1950	110	169.2	0.6	168.1	170.3	141	167.6	0.5	166.6	168.6
1960	51	171.3	0.9	169.4	173.2	62	169.2	0.8	167.7	170.7
1970	8	174.2	2.5	168.3	180.1	3	167.0	4.4	148.2	185.7

Source: AMHDB.

**Table 8:** Regression results. Determinants of height in the study area, birth cohorts 1890s–1970s.

		Dependent Variable: Height at 21 Years (Min. 1300 mm–Max. 1950 mm)					
Variable	Categories	(1)	(2)	(3)	(4)	(5)	(6)
Controllers	No (ref.)						
	Yes	10.11 *** (2.67)	16.53 *** (2.74)	9.81 *** (2.67)	9.85 *** (2.67)	13.10 *** (2.96)	10.96 *** (2.83)
Father’s occupation	Farmer (ref.)						
	Low skilled worker		−8.01 * (3.41)	−5.65 ** (3.40)	−5.63 * (3.40)	−8.01 ** (3.40)	−5.63 * (3.40)
	Artisan		1.50 (6.69)	2.34 (6.45)	2.44 (6.44)	0.33 (6.68)	2.70 (6.45)
	Upper class		29.56 *** (9.36)	21.71 ** (10.47)	21.32 ** (10.46)	29.31 *** (9.33)	20.59 ** (10.47)
	Other or unknown		−13.45 *** (4.03)	−3.01 (3.99)	−3.78 (3.99)	−13.10 *** (4.02)	−3.67 (3.99)
Literacy	Illiterate (ref.)						
	Literate		14.77 ** (6.29)	1.98 (6.12)	1.50 * (6.11)	12.38 * (6.30)	1.92 (6.12)
	Unknown		−16.05 (12.00)	−11.53 (11.43)	−11.95 (11.41)	−15.22 (11.96)	−12.10 (11.41)
Exemption appeals	No (ref.)						
	Physical				−12.14 *** (3.92)	−14.58 *** (4.11)	−12.06 *** (3.92)
	Social				8.34 (7.72)	−11.55 (7.82)	8.56 (7.72)
Number of living siblings (>5 years)	0–1 (ref.)						
	2–3					−10.56 ** (3.67)	−0.30 (3.55)
	4–6					−13.79 *** (3.95)	1.46 (3.96)
	7+					−10.41 ** (5.01)	7.22 (5.06)
	Intercept	1629.5 *** (6.90)	1632.1 *** (6.78)	1646.1 *** (9.03)	1635.0 *** (9.09)	1661.0 *** (7.63)	1632.2 *** (9.90)
Village fixed-effects		YES	NO	YES	YES	NO	YES
Birth decade fixed-effects		YES	NO	YES	YES	NO	YES
	Sample size	2510	2510	2510	2510	2510	2510
	Adjusted R^2^	0.144	0.150	0.037	0.154	0.047	0.155

Note: OLS estimates. * Statistical significance at 10%, ** statistical significance at 5%, *** statistical significance at 1%. Source: AMHDB.

**Table 9:** Average height (cm) at age 21 based on the number of grandparents, uncles and aunts living in the same locality at the time of birth, birth cohorts 1890–1969.

	1890–1969	1890–1909	1910–1929	1930–1949	1950–1969
0–2 people	165.4	163.6	164.0	166.4	168.8
3–5 people	165.9	163.7	164.5	166.6	169.1
6 or more people	167.0	162.6	165.9	168.1	169.1

Source: AMHDB. *n* = 1921 individuals.

## Data Availability

The data presented in this study are openly available at http://dx.doi.org/10.17632/ycpx8c8nvh.1 (Mendeley), accessed on 5 August 2021.

## Appendix A

**Table A1:** Percentage of individuals (distributed by height terciles) who controlled their fertility by stopping, by year of birth, 1870–1969.

	1870–1969	1870–1899	1900–1919	1920–1939	1940–1969
Short	56.6	44.7	56.6	57.2	62.3
Medium	56.4	44.4	55.7	62.7	50.8
Tall	51.3	32.3	51.4	55.3	52.9

Source: AMHDB. *n* = 1102. Note: The distribution by terciles has been calculated for each decade of birth.

**Table A2:** Mean age at first marriage of parents by tertile of height and year of birth, 1870–1959.

	1870–1959	1870–1899	1900–1929	1930–1959
Short	26.4	25.5	26.1	27.2
Medium	26.5	25.9	26.4	26.9
Tall	26.1	25.0	25.9	26.8

Source: AMHDB. *n* = 1198. Note: The distribution by tertiles has been calculated for each decade of birth.

**Table A3:** Percentage distribution of total individuals by number of grandparents and uncles living in the same locality, whether their parents controlled their fertility and year of birth, birth cohorts 1890–1969.

Controllers	1890–1969	1890–1909	1910–1929	1930–1949	1950–1969
0–2 people	43.6	38.8	44.3	43.7	44.1
3–5 people	43.7	53.1	40.1	44.0	44.1
6 or more people	12.7	8.1	15.6	12.3	11.8
Others	1890–1969	1890–1909	1910–1929	1930–1949	1950–1969
0–2 people	44.5	54.2	43.3	43.2	35.3
3–5 people	45.0	40.4	46.1	43.8	51.9
6 or more people	10.5	5.4	10.6	13.0	12.8

Source: AMHDB. *n* = 1921 individuals.

**Table A4:** Evolution of the average height, standard deviation and 95% confidence intervals of conscripts in the study area according to decade of birth, 1890–1979. These data correspond with Figure A1 (Appendix A).

Decade	Observation	Average	Standard Deviation	95% Confidence Interval	
1890	113	163.7	0.5	162.7	164.8
1900	312	163.7	0.3	163.1	164.4
1910	300	164.3	0.4	163.6	165.1
1920	561	164.5	0.3	164.0	165.0
1930	463	166.4	0.3	166.0	167.0
1940	386	167.5	0.3	167.0	168.1
1950	251	168.3	0.4	167.6	169.1
1960	113	170.1	0.6	169.0	171.3
1970	11	172.2	2.3	167.2	177.4

Source: AMHDB.

**Table A5:** Average height, standard deviation and 95% confidence intervals of agricultural labourers’ fathers, according to whether they controlled fertility by stopping or not, birth cohorts 1870–1969. These data correspond with Figure A2 (Appendix A).

	Controllers					Others				
Decade	Observation	Average	Standard Deviation	95% Confidence Interval		Observation	Average	Standard Deviation	95% Confidence Interval	
1870	4	164.1	1.6	158.9	169.4	11	164.2	1.1	161.8	166.6
1880	8	161.1	1.8	156.8	165.4	10	164.1	2.5	158.6	169.7
1890	16	162.3	1.5	159.2	165.4	6	163.5	1.9	158.7	168.4
1900	48	165.1	0.8	163.5	166.7	19	165.3	1.5	162.3	168.5
1910	23	165.8	1.3	163.1	168.6	28	164.7	1.2	162.3	167.2
1920	63	165.2	0.6	164.1	166.3	39	165.9	1.1	163.7	168.1
1930	44	167.9	0.9	166.1	169.8	32	166.2	0.9	164.4	168.0
1940	19	164.9	1.0	162.7	167.1	25	167.5	0.9	165.5	169.4
1950	23	168.6	1.1	166.4	170.8	22	168.2	1.1	165.8	170.6
1960	10	169.7	1.7	165.8	173.6	3	174.3	2.3	164.3	184.3

Source: AMHDB.

**Table A6:** Average height, standard deviation and 95% confidence intervals of husbandmen’ fathers, according to whether they controlled fertility by stopping or not, birth cohorts 1870–1969. These data correspond with Figure A3 (Appendix A).

	Controllers					Others				
Decade	Observation	Average	Standard Deviation	95% Confidence Interval		Observation	Average	Standard Deviation	95% Confidence Interval	
1870	-	161.0	-	-	-	7	162.2	2.1	157.1	167.3
1880	2	160.2	0.2	157.1	163.4	-	160.5	-	-	-
1890	-	161.0	-	-	-	-	164.0	-	-	-
1900	8	160.9	0.6	159.5	162.3	12	164.2	1.1	161.8	166.7
1910	11	166.0	2.1	161.3	170.7	16	165.2	1.9	161.3	169.1
1920	29	165.6	1.0	163.7	167.6	15	166.0	1.6	162.6	169.4
1930	32	166.4	1.0	164.4	168.5	17	168.5	1.3	165.8	171.3
1940	20	168.9	1.0	166.8	171.1	18	170.5	1.2	167.9	173.0
1950	14	169.0	1.0	166.8	171.3	8	170.5	1.8	166.1	174.8
1960	4	175.5	2.3	168.1	182.9	7	170.0	1.5	166.2	173.8

Source: AMHDB.

**Table A7:** Average height, standard deviation and 95% confidence intervals of children of agricultural labourers, according to whether or not their parents controlled their fertility by stopping, birth cohorts 1890–1979. These data correspond with Figure A5 (Appendix A).

	Controllers					Others				
Decade	Observation	Average	Standard Deviation	95% Confidence Interval		Observation	Average	Standard Deviation	95% Confidence Interval	
1890	6	162.0	2.5	155.7	168.4	53	162.6	0.8	161.0	164.2
1900	19	165.1	1.2	162.6	167.6	133	164.4	0.5	163.3	165.5
1910	28	165.1	1.2	162.7	167.5	123	163.4	0.6	162.1	164.7
1920	88	164.8	0.7	163.4	166.3	173	164.0	0.5	163.0	164.9
1930	104	167.0	0.5	166.0	168.1	147	165.5	0.5	164.4	166.6
1940	82	167.7	0.7	166.3	169.2	146	166.5	0.5	165.5	167.5
1950	56	169.2	0.8	167.5	170.9	88	167.5	0.7	166.1	168.8
1960	26	170.7	1.4	167.9	173.6	35	168.8	1.0	166.7	170.9
1970	4	172.7	5.0	156.7	188.7	1	160.0	-	-	-

Source: AMHDB.

**Table A8:** Average height, standard deviation and 95% confidence intervals of children of husbandmen, according to whether or not their parents controlled their fertility by stopping, birth cohorts 1890–1979. These data correspond with Figure A6 (Appendix A).

	Controllers					Others				
Decade	Observation	Average	Standard Deviation	95% Confidence Interval		Observation	Average	Standard Deviation	95% Confidence Interval	
1890	1	162.0	-	-	-	10	163.4	1.6	159.7	167.0
1900	12	165.5	1.5	162.3	168.8	22	161.9	1.1	159.6	164.3
1910	9	165.8	2.5	160.0	171.5	50	165.0	1.0	163.1	167.0
1920	25	166.0	1.4	163.0	169.0	77	165.4	0.6	164.2	166.6
1930	50	167.1	0.9	165.3	168.8	72	165.8	0.7	164.5	167.1
1940	35	168.8	1.3	166.1	171.5	49	168.3	0.8	166.7	169.8
1950	23	170.0	1.0	168.1	172.0	19	168.7	1.3	165.9	171.5
1960	12	170.0	1.7	166.2	173.8	8	168.6	2.0	163.8	173.4
1970	1	174.0	-	-	-	1	166.0	-	-	-

Source: AMHDB.
